# Pan-metastatic cancer analysis of prognostic factors and a prognosis-based metastatic cancer classification system

**DOI:** 10.18632/aging.103467

**Published:** 2020-08-27

**Authors:** Chao Zhang, Guijun Xu, Yao Xu, Haixiao Wu, Xu Guo, Min Mao, Vladimir P. Baklaushev, Vladimir P. Chekhonin, Karl Peltzer, Ye Bai, Guowen Wang, Wenjuan Ma, Xin Wang

**Affiliations:** 1 Department of Bone and Soft Tissue Tumours, Tianjin Medical University Cancer Institute and Hospital, National Clinical Research Center for Cancer, Key Laboratory of Cancer Prevention and Therapy, Tianjin’s Clinical Research Center for Cancer, Tianjin 300060, China; 2 Department of Orthopedics, Tianjin Hospital, Tianjin 300060, China; 3 Department of Orthopedics, Cangzhou Central Hospital, Cangzhou 061000, Hebei Province, China; 4 Department of Pathology and Southwest Cancer Center, First Affiliated Hospital, Army Medical University, Chongqing 400038, China; 5 Federal Research and Clinical Center of Specialized Medical Care and Medical Technologies, Federal Biomedical Agency of the Russian Federation, Moscow 115682, Russian Federation; 6 Department of Basic and Applied Neurobiology, Federal Medical Research Center for Psychiatry and Narcology, Moscow 117997, Russian Federation; 7 Department of Research and Innovation, University of Limpopo, Turfloop 0527, South Africa; 8 Department of Epidemiology and Health Statistics, School of Public Health and Management, Chongqing Medical University, Chongqing 400038, China; 9 Department of Breast Imaging, Tianjin Medical University Cancer Institute and Hospital, National Clinical Research Center for Cancer, Key Laboratory of Cancer Prevention and Therapy, Tianjin’s Clinical Research Center for Cancer, Tianjin 300060, China; 10 Department of Health Management Center (Epidemiology and Biostatistics), First Affiliated Hospital, Army Medical University, Chongqing 400038, China

**Keywords:** neoplasm metastasis, prognosis, cancer classification, SEER program

## Abstract

We aimed to perform a pan-metastatic cancer analysis on survival and prognostic factors and to create a prognosis-based classification system. We selected distant metastasis patients from the Surveillance, Epidemiology, and End Results (SEER) database. The associations between the characteristics of the patients at admission and overall survival were determined. A prognosis-based metastatic cancer classification was established based on the identified prognostic factors. The differences in prognosis among these categories were tested. The survival rate and prognostic factors were not consistent across cancers. Three metastatic cancer categories were generated, each with different prognoses. The prognostic differences among the categories were satisfactorily validated. Different metastatic cancer types had homogeneous and heterogeneous survival rates and prognostic factors. A prognosis-based classification system for synchronous distant metastasis cancer patients at admission was created. This classification system reflects the grade of malignancy in metastatic cancers and may guide the prediction of survival and individualized treatment. Moreover, it may have important implications for the management of synchronous metastatic cancers and aid clinicians in properly allocating medical resources to metastatic patients.

## INTRODUCTION

Decades of cancer research and clinical trials have revealed genetic, epidemiological, and anatomical characteristics that have led to the development of plausible therapeutic strategies, many of which have significantly improved clinical outcomes [1]. Based on the TNM classification, physicians can conveniently predict the prognosis of cancer patients, select appropriate treatment regimens, and improve the efficiency of clinical treatment [2, 3]. It is well known that distant metastasis (DM) is the main characteristic of stage IV cancer, and it accounts for 90% of cancer-related deaths in patients with clinical symptoms [4].

The prognosis of cancer patients is one of the primary factors guiding treatment. However, there has been no classification system developed to predict the prognosis of patients with DM. The anatomical system may be an excellent choice for predicting the prognosis of metastatic cancers, as they may share common pathogenic mechanisms and present similar symptoms. However, a large number of studies have suggested that different types of metastatic cancers showed both homogeneous and heterogeneous prognoses, even in the same anatomical system [5, 6]. Genetics may be another approach to identify the differences in survival among metastatic cancers. However, weaknesses of this approach, including the high cost, complex detection process, and extended detection period, have resulted in the limited application of genetic techniques in the clinic. In our recently published papers, a series of factors were found to contribute to the prognosis of metastatic cancers. The identified factors provided a basis for constructing a metastatic cancer classification system [7–11].

Based on the previously identified prognostic factors, several systems for the evaluation of survival in patients with stage IV cancer have been widely used in different fields, such as the Diagnosis-Specific Graded Prognostic Assessment (DS-GPA) for brain metastasis [12], Tokuhashi score and Tomita score for spinal metastasis [13], and Glasgow prognostic score (GPS) for liver metastasis [14]. However, due to the limited sample size and the relatively limited cancer types, the external applicability of these tools is not satisfactory [15]. These classification tools cannot be used to distinguish the differences in survival of patients with cancers in the same or different anatomical systems.

The Surveillance, Epidemiology and End Results (SEER) database consists of 18 population-based cancer registries and has recorded DM since 2010. To date, the SEER database has recorded more than sixty cancer types and incorporated more than 10 million patients. Thus, the present study aimed to evaluate the differences among the characteristics, survival and prognostic factors in all patients with metastatic cancers, to construct a prognosis-based pan-metastatic cancer classification system, to support the implementation of different metastatic cancer management strategies and to guide physicians in the selection of individualized treatment regimens for stage IV cancer patients.

## RESULTS

### Characteristics of the included participants

A total of 291,104 metastatic cancer patients with cancer in 61 sites were included in the construction cohort in the present study. In these patients, the mean age was 67.12±13.40 years (0-113 years), 52.6% (N=153,228) were male, and 51.7% were married (N=142,757). Most of the patients were white (N=230,342, 79.3%), and 80.1% of them were insured (N=227,272). The demographic and clinical characteristics stratified by cancer site are described in Figure 1.

**Figure 1:**
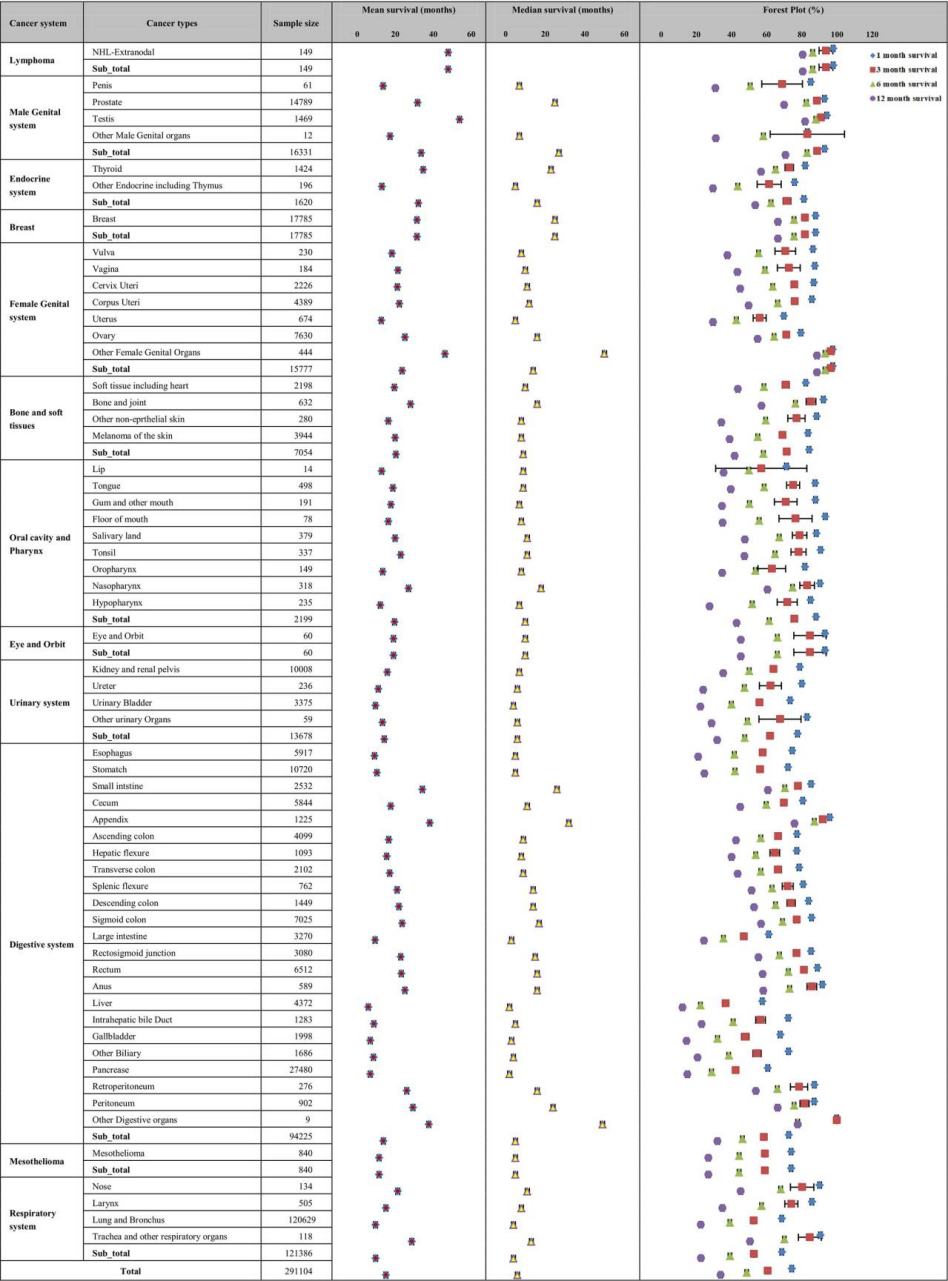
Distribution of demographic (**A**) and clinical characteristics (**B**) for the included patients in the construction cohort. The figure describes the distributions of the demographic characteristics of age, sex, marital status, insurance status and race and the clinical factors of organ metastases; grade; T, N, and M stages; and surgery status among the 61 included metastatic cancer types.

**Figure 1:**
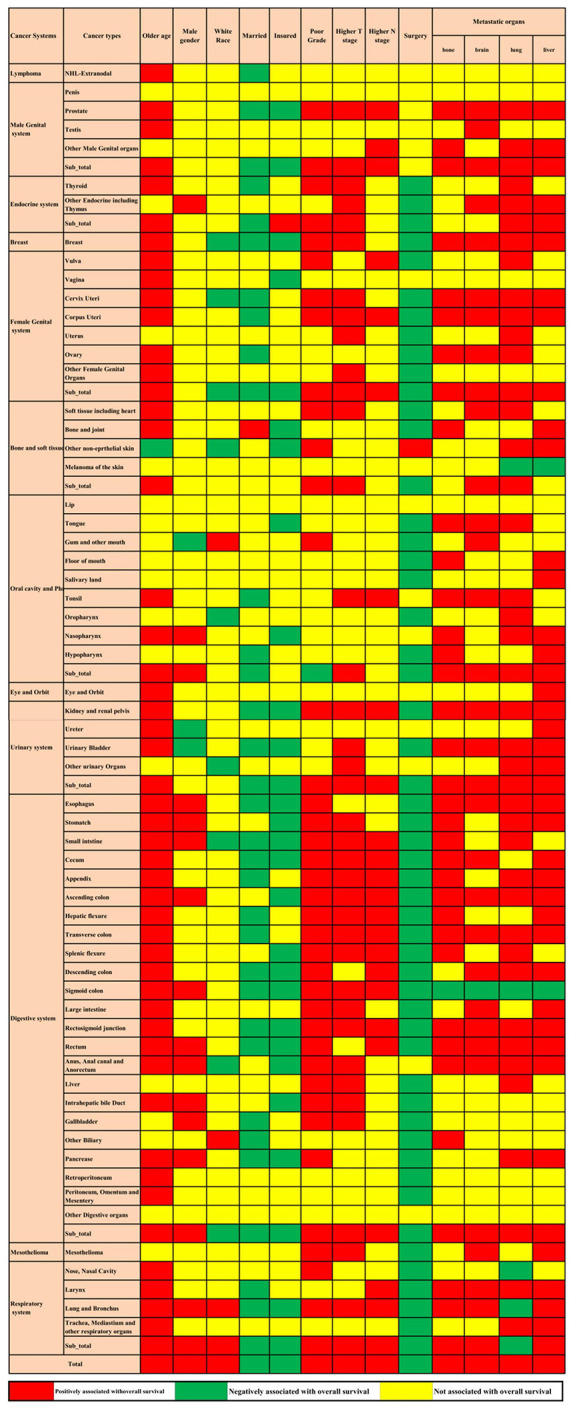
Distribution of demographic (**A**) and clinical characteristics (**B**) for the included patients in the construction cohort. The figure describes the distributions of the demographic characteristics of age, sex, marital status, insurance status and race and the clinical factors of organ metastases; grade; T, N, and M stages; and surgery status among the 61 included metastatic cancer types.

A total of 252,535 metastatic cancer patients were included in the validation cohort. The mean age was 66.94±13.44 years, and 52.9% were males (N=133,486). The demographic and clinical characteristics were comparable between the construction and validation cohorts. However, due to the relatively large sample size of the participants, significant differences existed (Table 1).

**Table 1:** The difference in the demographic and clinical characteristics between construction and validation cohort of metastatic cancer patients in SEER.

**Factors**	**Construction cohort N(%)**	**Validation cohort N(%)**	**Chi-square**	***P-value***
**Age**			63.77	*<0.001*
≤60	86819(29.8)	77836(30.8)		
>60	204285(70.2)	174699(69.2)		
**Sex**			2.66	*0.10*
Female	137876(47.4)	119049(47.1)		
Male	153228(52.6)	133486(52.9)		
**Race**			107.58	*<0.001*
Others	60119(20.7)	49351(19.6)		
White	230342(79.3)	202890(80.4)		
**Marriage**			101.11	*<0.001*
Married	142757(51.7)	129092(53.1)		
Unmarried	133517(48.3)	114163(46.9)		
**Insurance**			10.25	*<0.001*
Uninsured	11946(4.2)	6325(4.2)		
Any medical aid	44366(15.6)	21482(14.3)		
Insured	227272(80.1)	122475(81.5)		
**T stage**			47.86	*<0.001*
T1	30010(13.7)	25287(13.4)		
T2	52661(24.0)	41294(21.8)		
T3	68528(31.2)	42672(22.6)		
T4	68625(31.2)	79868(42.2)		
**N stage**			11.63	*<0.001*
N0	96485(38.5)	74606(37.0)		
N1	60656(24.2)	46957(23.3)		
N2	67197(26.8)	61029(30.2)		
N3	26103(10.4)	19200(9.5)		
**Grade**			8.88	*<0.001*
Grade I	7950(6.0)	6378(5.2)		
Grade II	42621(32.10	37862(31.2)		
Grade III	68797(51.9)	64892(53.4)		
Grade IV	13264(10.0)	12378(10.2)		
**Surgery**			573.04	*<0.001*
No	236836(81.7)	198630(79.1)		
Yes	53119(18.3)	52502(20.9)		

### Overall survival of different metastatic cancers

**Figure 2:**
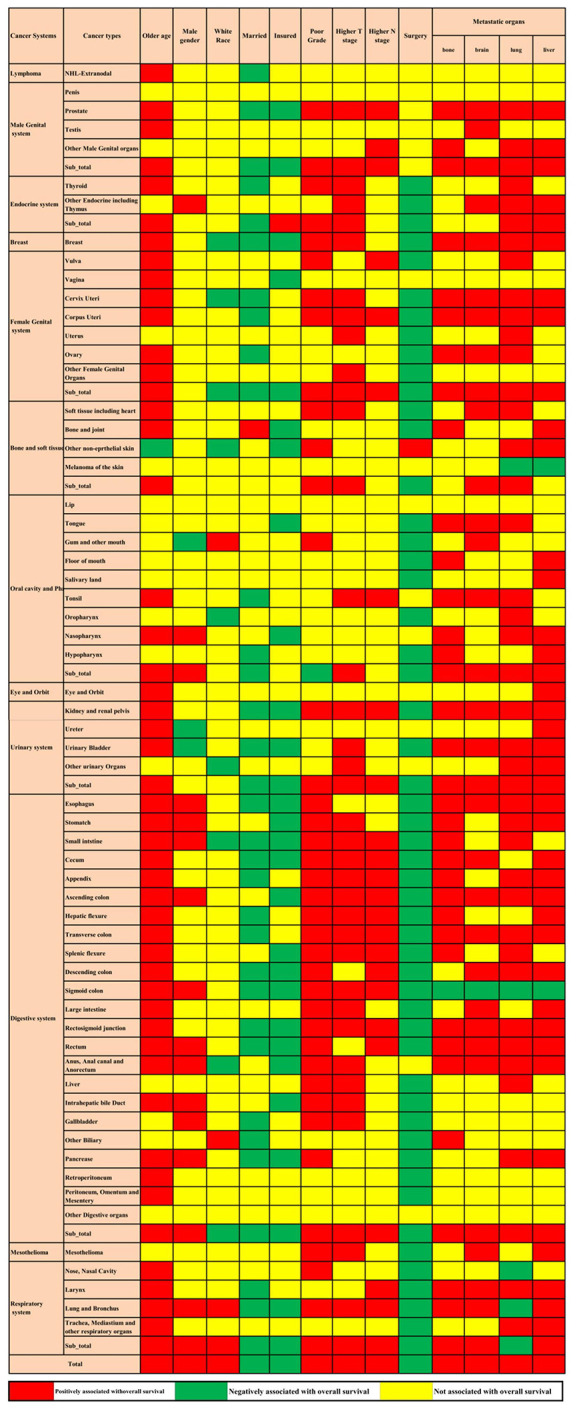
**Mean and median survival times and survival rates for the 61 metastatic cancer types in the construction cohort.** The figure describes the mean and median survival for the metastatic cancer types and cancer systems as box plots, and the 1-month, 3-month, 6-month and 12-month survival rates with 95% CIs are also shown in the forest plot.

In total, the mean and median survival times of the metastatic cancer patients in the construction cohort were 15.20 (95% CI: 15.12-15.28) months and 6.00 (95% CI: 5.95-6.06) months, respectively. The 1-, 3-, 6- and 12-month survival rates were 74.4%, 60.8%, 48.8% and 34.0%, respectively (Figure 2).

The survival rate and survival time were not consistent across cancers in different systems. DM patients with primary cancer in the respiratory system exhibited the lowest mean survival time (9.80±0.05 months) and 12-month survival rate (22.8%). DM patients with primary cancer in the lymphoma system had the highest mean survival time (47.90±2.33 months), while the female genital system had the highest 12-month survival rate (88.8%).

For different cancer types, the prognosis was not consistent. Metastatic liver cancer (mean survival time: 5.89±0.18 months; 12-month survival rate: 12.3%), gallbladder cancer (mean survival time: 6.95±0.27 months; 12-month survival rate: 14.6%) and pancreatic cancer (mean survival time: 7.00±0.08 months; 12-month survival rate: 15.1%) had the shortest survival times and lowest survival rates of all cancer sites. Metastatic testicular cancer had the highest mean survival time of 54.0±0.75 months, but metastatic carcinoma of the female genital system had the highest 12-month survival rate (88.8%).

### Prognostic factors for different metastatic cancers

**Figure 3:**
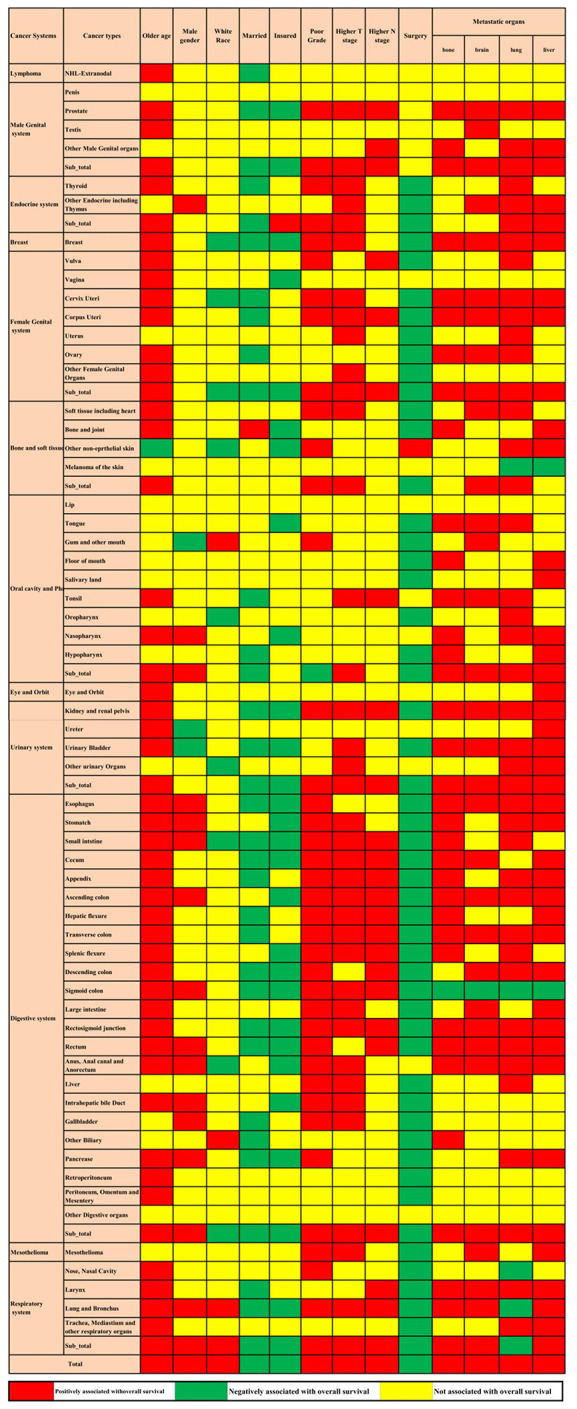
**Prognostic factors for the 61 metastatic cancer types in the construction cohort.** The red colour and green colour describe risk factors and protective factors for the survival of metastatic cancers, respectively, while the yellow colour indicates that the factor did not reach the significance level.

Multivariable Cox regression showed that advanced age, male sex, white race, poorly differentiated grade, higher T stage, higher N stage, and bone, brain, lung, and liver metastases were all positively associated with overall mortality. Married status, insured status, and surgery at the primary site were all negatively related to overall mortality. The associations between the factors mentioned above and overall survival were not consistent across cancer in different systems and cancer types. These factors were all associated with metastatic lung and bronchus cancer; however, metastatic cancers of other digestive organs and the penis were not associated with any of these factors. Even in the same system, the factors associated with metastatic cancer in different sites were not consistent (Figure 3).

### Prognosis-based metastatic cancer classification

Unsupervised hierarchical clustering analysis was used to classify the 61 cancer sites into three main subgroups, namely, categories A, B, and C. The category A metastatic cancer subgroup had the worst prognosis and included intrahepatic bile duct cancer, stomach cancer, oesophageal cancer, urinary bladder cancer, other biliary cancer, lung and bronchus cancer, mesothelioma, another endocrine including thymus cancer, uterus cancer, ureter cancer, lip cancer, liver cancer, pancreatic cancer, gallbladder cancer, and large intestine cancer (Figure 4A). With the best prognosis, the category C metastatic cancer subgroup included metastatic NHL-extranodal cancer, testis cancer, other female genital organ cancer, appendix cancer, prostate cancer, and other digestive organ cancers. Details about the categories across different anatomical systems are provided in the Supplementary Table 1.

**Figure 4:**
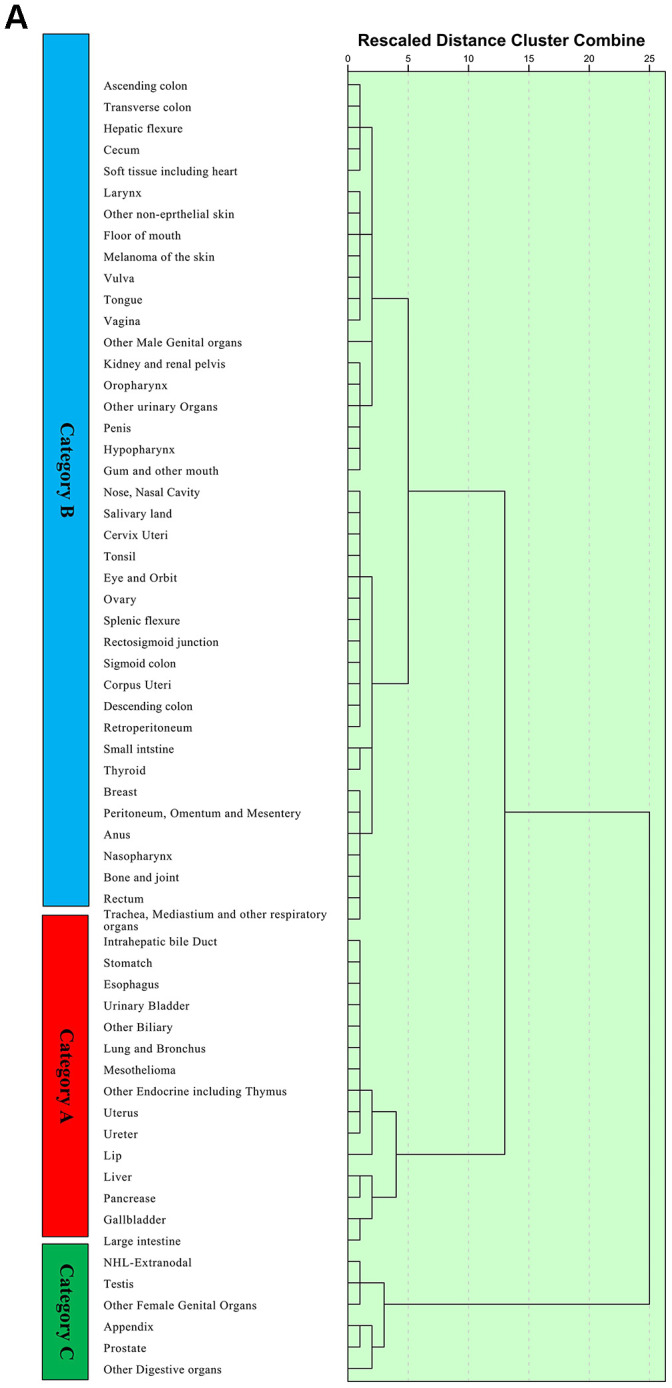
Unsupervised hierarchical cluster analysis for the classification of metastatic cancer types (**A**) and the differences in survival among these three categories in the construction cohort (**B**) and validation cohort (**C**). All 61 metastatic cancer types were sub-grouped into three categories, namely, categories (**A**–**C**) and the Kaplan-Meier analysis suggested that there were significant differences in prognoses among these categories. Additionally, the survival differences among these categories were validated in the validation cohort.

**Figure 4:**
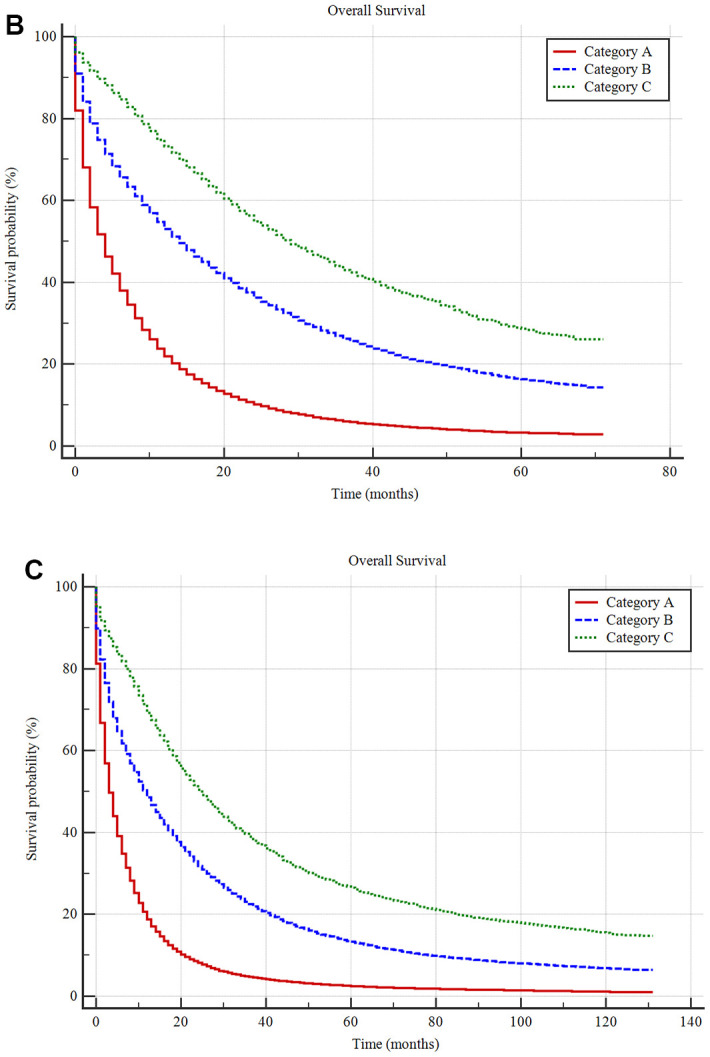
Unsupervised hierarchical cluster analysis for the classification of metastatic cancer types (**A**) and the differences in survival among these three categories in the construction cohort (**B**) and validation cohort (**C**). All 61 metastatic cancer types were sub-grouped into three categories, namely, categories (**A**–**C**) and the Kaplan-Meier analysis suggested that there were significant differences in prognoses among these categories. Additionally, the survival differences among these categories were validated in the validation cohort.

The Kaplan-Meier method showed that the mean survival times for the A, B, and C metastatic cancer subgroups were 9.24±0.04 months (median survival 4.00±0.02 months), 23.43±0.09 months (median survival 14.00±0.10 months) and 34.60±0.22 months (median survival 27.00±0.36 months), respectively, with a significant difference among them (*P <0.001*) (Figure 4B).

For the validation cohort in the SEER database, the mean survival times in the A, B, and C metastatic cancer subgroups were 9.47±0.05 months (median survival 4.00±0.02 months), 27.22±0.13 months (median survival 12.00±0.10 months) and 44.55±0.38 months (median survival 26.00±0.37 months), respectively. There was a significant difference among the three categories (*P <0.001*) (Figure 4C).

## DISCUSSION

In this study, a comprehensive pan-metastatic cancer analysis was conducted to evaluate survival and to identify prognostic factors for stage IV cancer. Significantly different metastatic cancers had distinct prognoses, even in the same anatomical system. Metastatic respiratory system cancers had the shortest mean survival time, while metastatic female genital system cancers had the longest survival time. The median survival time and 12-month survival rate of patients with stage IV cancer were six months and 34.0%, respectively. For DM patients who may benefit from treatment, individualized treatment plans should be carefully formulated based on the significantly different prediction of prognosis according to the primary cancer and the metastatic site. The present study can be the foundation for the formulation of an individualized evaluation system for stage IV cancer.

For the first time, based on a large population from the SEER database, we summarized all the prognostic factors in various systems and cancer types for stage IV cancer. The identification of prognostic factors in stage IV cancer patients is a major concern in the DM screening and individualized treatment. In the present study, advanced age, male sex, white race, poorly differentiated grade, higher T stage, higher N stage, and bone, brain, lung, and liver metastases were positively associated with overall mortality. Married status, insured status, and surgery at the primary site were all negatively associated with overall mortality. Previously, some prognostic factors in certain cancers were reported [16–18]. The latest study, based on a single-centre population, reported that extracranial metastases and Karnofsky performance status were independent prognostic factors in colorectal cancer patients with brain metastasis [19]. Another study focused on bone metastases of hepatocellular carcinoma reported a series of prognostic factors, including Child-Pugh class A group, alpha-fetoprotein level more than 30 ng/mL, and higher T stage (>5 cm) [20]. Based on 202 lung cancer patients with bone metastasis, another study reported that age (<60 years), non-small-cell lung cancer pathology type, chemotherapy for bone metastasis, and radiation therapy for bone metastasis were independent favourable prognostic factors [21]. Thus, as indicated by our results in each system and cancer type (Figure 3), the prognostic factors are both homogeneous and heterogeneous. To precisely predict the survival of stage IV cancer patients, studies identifying specific prognostic factors in different stage IV cancers should be performed.

In addition, based on the survival analysis in the pan-metastatic cancer cohort, we initially classified all cancers with DM into three subgroups. To the best of our knowledge, the present classification is the first pan-metastatic cancer prognosis-based system for stage IV cancer. Currently, TNM staging has been widely accepted as one of the main tools for evaluating cancer patients. With medical developments and improved survival in cancer patients, the number of patients with DM has been increasing. The present study suggests that there are different survival rates in various cancers with DM, which is supported by evidence from previous studies [5, 22]. Thus, among cancer patients in the M1 stage, limited guidance can be provided by the TNM stage regarding the selection of the appropriate treatment. Further classification of patients with M1 stage disease is warranted. Currently, to predict the survival of cancer patients with stage IV disease, most physicians and researchers have classified patients based on the anatomical system. However, such classification was proven to be inaccurate in the present study. We hypothesize that different histological types of cancer are heterogeneous within the same anatomical system or even within the same cancer type. Different histological types may have different prognoses [23–25]. In the present study, the constructed classification system was shown to reflect the grade of malignancy of metastatic cancer and may offer important survival information that can be used to guide the formulation of a survival prediction scoring system and treatment selection for stage IV cancer patients.

Synchronous metastasis was accepted as the diagnosis of a distant metastasis with the primary cancer. Metachronous metastasis was usually defined as an occurrence after a period post treatment. Previously, patients with synchronous metastasis, compared with those with metachronous metastasis, have more adverse prognostic features, significantly shorter time to treatment failure, and poorer survival [26]. In the latest study, timing of metastases after initial diagnosis impacts outcome from targeted therapy in cancer [26]. However, seldom study was performed to reveal the potential mechanism under the differences between the synchronous metastasis and metachronous metastasis. Thus, more studies and trials are needed in future.

At the same time, with the increase in the therapy costs of cancer, issues related to medical resource allocation and medical insurance decisions have become global concerns [27, 28]. The constructed classification system can help medical officials in the metastatic cancer management and in the distribution of medical resources for stage IV cancer patients. In addition, with the identified prognostic factors for all cancers, the value of treatment options for metastatic cancer can be considered when medical insurance policies are generated.

For these three different classifications, only the distribution of the association between male sex and overall survival was significantly different among categories A, B, and C (Table 2). However, we did not find any obvious rules for the other prognostic factors in different categories. This may be explained by the fact that this metastatic cancer classification system was only based on the prognosis of the cancers, not the pathogenesis.

**Table 2:** The differences in the distribution of the associations of the potential factors and overall survival among categories A, B, and C.

**Prognostic factors**	**categories A**	**categories B**	**categories C**	**Chi-square**	***P-value***
**Older age**				3.42	0.49
Not significant	7(46.7)	10(25.0)	1(18.7)		
Negatively	0(0.0)	1(2.5)	0(0.0)		
Positively	8(53.3)	29(72.5)	5(83.3)		
**Male gender**				12.77	0.01
Not significant	6(40.0)	33(82.5)	6(100.0)		
Negatively	2(13.3)	1(2.5)	0(0.0)		
Positively	7(46.7)	6(15.0)	0(0.0)		
**White race**				6.87	0.14
Not significant	13(86.7)	32(80.0)	6(100.0)		
Negatively	0(0.0)	7(17.5)	0(0.0)		
Positively	2(13.3)	1(2.5)	0(0.0)		
**Married status**				0.71	0.95
Not significant	9(60.0)	22(55.0)	3(50.0)		
Negatively	6(40.0)	17(42.5)	3(50.0)		
Positively	0(0.0)	1(2.5)	0(0.0)		
**Insurance**				1.25	0.53
Not significant	9(60.0)	24(60.0)	5(83.3)		
Negatively	6(40.0)	16(40.0)	1(16.7)		
**Poor Grade**				1.22	0.54
Not significant	6(40.0)	19(47.5)	4(66.7)		
Positively	9(60.0)	21(52.5)	2(33.3)		
**Higher T stage**				2.55	0.28
Not significant	5(33.3)	23(57.5)	3(50.0)		
Positively	10(66.7)	17(42.5)	3(50.0)		
**Higher N stage**				5.67	0.06
Not significant	14(93.3)	24(60.0)	4(66.7)		
Positively	1(6.7)	16(40.0)	2(33.3)		
**Surgery**				7.37	0.12
Not significant	2(13.3)	9(22.5)	4(66.7)		
Negatively	13(86.7)	30(75.0)	2(33.3)		
Positively	0(0.0)	1(2.5)	0(0.0)		
**Bone metastasis**				3.47	0.48
Not significant	10(66.7)	17(42.5)	4(66.7)		
Negatively	0(0.0)	1(2.5)	0(0.0)		
Positively	5(33.3)	22(50.0)	2(33.3)		
**Brain metastasis**				0.78	0.94
Not significant	9(60.0)	22(55.0)	4(66.7)		
Negatively	0(0.0)	1(2.5)	0(0.0)		
Positively	6(40.0)	17(42.5)	2(33.3)		
**Lung metastasis**				3.82	0.43
Not significant	7(46.7)	12(30.0)	4(66.7)		
Negatively	1(6.7)	3(7.5)	0(0.0)		
Positively	7(46.7)	25(62.5)	2(33.3)		
**Liver metastasis**				2.75	0.60
Not significant	6(40.0)	15(37.5)	4(66.7)		
Negatively	0(0.0)	2(5.0)	0(0.0)		
Positively	9(60.0)	23(57.5)	2(33.3)		

There were some limitations of our study. First, DM was merely recorded in the bone, liver, lung, and brain in the SEER database. Metastasis to other sites, which may have resulted in a bias in the survival analysis, was not recorded. Second, the present study analysed the associations between overall survival and the characteristics of patients with synchronous metastasis at admission. The occurrence of metastasis during follow-up, namely, metachronous metastasis, was not investigated, and the results may have been affected. Thus, the results should be interpreted with caution, and more studies are needed to further validate their application. Third, because of the lack of detailed costs for the patients, the present study cannot further analyse the cost-effectiveness through the constructed classification based on the pan-metastatic cancer cohort. Moreover, due to the lack of a large cohort focused on DM in cancer patients, the validity of the prognosis-based classification system still needs to be further externally tested.

In summary, this nationwide, population-based study comprehensively analysed pan-metastatic cancer survival and identified prognostic factors in patients with all stage IV cancers at admission. The present study suggests that the survival of patients with synchronous distant metastasis is both homogeneous and heterogeneous. A series of prognostic factors in stage IV cancer patients were identified; advanced age, male sex, white race, poorly differentiated grade, higher T stage, higher N stage, and bone, brain, lung and liver metastases were positively associated with overall mortality. The prognostic factors in various systems and cancer types were both homogeneous and heterogeneous. Based on the different survival of stage IV cancer patients, all metastatic cancers were divided into three subgroups. This classification reflects the grade of malignancy of metastatic cancer and may offer important survival information that can be used to guide the formulation of a survival prediction system and the selection of appropriate treatments. Moreover, the constructed classification system can help medical officials manage synchronous distant metastatic cancers and properly allocate medical resources for stage IV cancer patients.

## MATERIALS AND METHODS

### Study population

**Figure 5:**
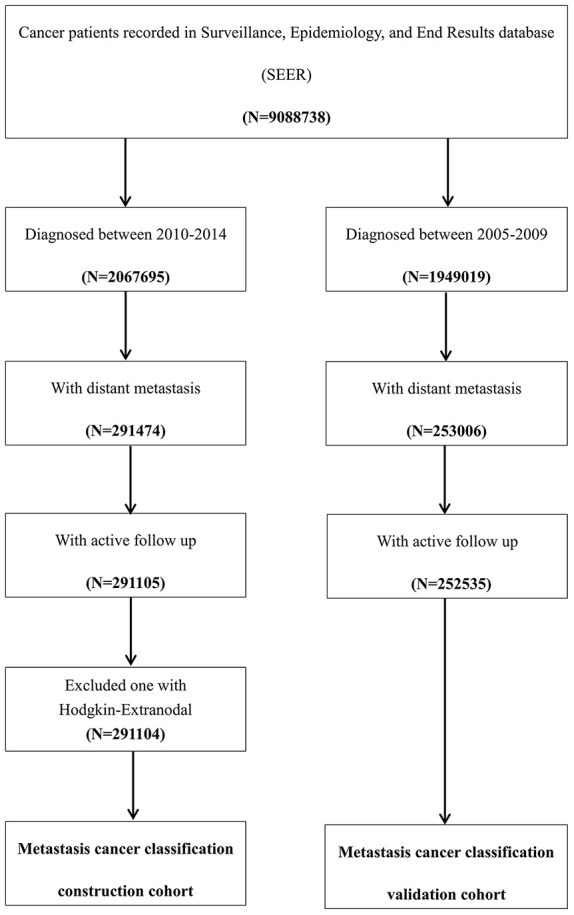
**Flow chart of the patient selection procedure in the construction and validation cohort.** Metastatic cancer patients who were diagnosed between 2010 and 2014 were included as the construction cohort, which was used to construct the metastatic cancer categories, and those who were diagnosed between 2005 and 2009 were included in the validation cohort, which was used to test the predictive accuracy of this classification system.

This study used a metastatic cancer case cohort derived from the National Cancer Institute SEER cohort. The SEER database covers approximately 30% of the total United States population. Patients with metastatic cancer according to the American Joint Committee on Cancer (AJCC) staging system, 7^th^ edition, who were diagnosed between 2010 and 2014 were included as the construction cohort in the present study. Patients with metastatic cancer who were diagnosed between 2005 and 2009 in the SEER cohort were included as the validation cohort. Patients who were diagnosed by death certificate or autopsy were excluded. SEER*Stat Software version 8.3.5 (https://seer.cancer.gov/seerstat/) (Information Management Service, Inc., Calverton, MD, USA) was used to generate the case list (Figure 5).

### Ethics statement

Cancer is a reportable disease in every state of the United States, and use of the data in the SEER database does not require informed patient consent. The present study complied with the 1964 Helsinki Declaration and its later amendments or comparable ethical standards.

### Statistical analysis

Normally distributed data, such as age, are described as the means ± standard deviations (SDs). The mean and median survival of the patients are described as the survival time with 95% confidence intervals (CIs). Categorical data, such as sex, are presented as numbers and percentages (N, %), and the differences between groups were tested by Pearson’s chi-square test or the rank-sum test. The Kaplan-Meier method was used to investigate the 1-, 3-, 6- and 12-month survival rates and the mean and median survival of patients with metastatic cancer at various sites. Univariable Cox regression was used to investigate the potential factors associated with the overall survival of the cancer patients, and the factors with *P*-values smaller than 0.1 were incorporated into the multivariable Cox regression model.

Unsupervised hierarchical clustering analysis was performed using the squared Euclidean distance method based on the patients’ demographic, clinical and prognostic features, including age; sex; race; marital status; insurance; differentiation grade; T stage; N stage; surgery; bone, brain, liver and lung metastases; 1-, 3-, 6-, and 12-month survival rates; and mean survival. Tree cluster analysis was performed to classify the metastatic cancer sites into categories A, B, and C. Kaplan-Meier analysis was performed to determine the prognosis of the category A, B, and C metastatic cancer subgroups, and differences were identified with the log-rank test. Moreover, metastatic cancer patients who were diagnosed between 2005 and 2009 were used for the validation of the classification system. Two-tailed *P*-values *<0.05* were statistically significant. Statistical analyses were performed using the Statistical Package for the Social Sciences (SPSS) version 23.0 software package for Windows (SPSS version 20.0, IBM, Inc.).

## Supplementary Material

Supplementary Table 1
