# Supplementary material for: Pan-metastatic cancer analysis of prognostic factors and a prognosis-based metastatic cancer classification system
Source: Aging (Albany NY). 2020 Aug 27;12(16):16046–61. doi: 10.18632/aging.103467 (PMC7485706; doi:10.18632/aging.103467)
Supplement: Supplementary Table 1 [file aging-12-103467-s001..docx]

Supplementary Table 1. Details about the categories across different anatomic systems.

| **Cancer Systems** | **Cancer types** | **Category** | | |
| --- | --- | --- | --- | --- |
|  |  | **A** | **B** | **C** |
| **Oral cavity and Pharynx** | **Lip** | **√** |  |  |
|  | **Tongue** |  | **√** |  |
|  | **Gum and other mouth** |  | **√** |  |
|  | **Floor of mouth** |  | **√** |  |
|  | **Salivary land** |  | **√** |  |
|  | **Tonsil** |  | **√** |  |
|  | **Oropharynx** |  | **√** |  |
|  | **Nasopharynx** |  | **√** |  |
|  | **Hypopharynx** |  | **√** |  |
| **Digestive system** | **Esophagus** | **√** |  |  |
|  | **Stomatch** | **√** |  |  |
|  | **Small intstine** |  | **√** |  |
|  | **Cecum** |  | **√** |  |
|  | **Appendix** |  |  | **√** |
|  | **Ascending colon** |  | **√** |  |
|  | **Hepatic flexure** |  | **√** |  |
|  | **Transverse colon** |  | **√** |  |
|  | **Splenic flexure** |  | **√** |  |
|  | **Descending colon** |  | **√** |  |
|  | **Sigmoid colon** |  | **√** |  |
|  | **Large intestine** | **√** |  |  |
|  | **Rectosigmoid junction** |  | **√** |  |
|  | **Rectum** |  | **√** |  |
|  | **Anus, Anal canal and Anorectum** |  | **√** |  |
|  | **Liver** | **√** |  |  |
|  | **Intrahepatic bile Duct** | **√** |  |  |
|  | **Gallbladder** | **√** |  |  |
|  | **Other Biliary** | **√** |  |  |
|  | **Pancrease** | **√** |  |  |
|  | **Retroperitoneum** |  | **√** |  |
|  | **Peritoneum, Omentum and Mesentery** |  | **√** |  |
|  | **Other Digestive organs** |  |  | **√** |
| **Respiratory  system** | **Nose, Nasal Cavity** |  | **√** |  |
|  | **Larynx** |  | **√** |  |
|  | **Lung and Bronchus** | **√** |  |  |
|  | **Trachea, Mediastium and other respiratory organs** |  | **√** |  |
| **Bone and soft tissues** | **Soft tissue including heart** |  | **√** |  |
|  | **Bone and joint** |  | **√** |  |
|  | **Other non-eprthelial skin** |  | **√** |  |
|  | **Melanoma of the skin** |  | **√** |  |
| **Breast** | **Breast** |  | **√** |  |
| **Female Genital  system** | **Vulva** |  | **√** |  |
|  | **Vagina** |  | **√** |  |
|  | **Cervix Uteri** |  | **√** |  |
|  | **Corpus Uteri** |  | **√** |  |
|  | **Uterus** | **√** |  |  |
|  | **Ovary** |  | **√** |  |
|  | **Other Female Genital Organs** |  | **√** |  |
| **Male Genital  system** | **Penis** |  | **√** |  |
|  | **Prostate** |  |  | **√** |
|  | **Testis** |  |  | **√** |
|  | **Other Male Genital organs** |  |  | **√** |
| **Urinary system** | **Kidney and renal pelvis** |  | **√** |  |
|  | **Ureter** | **√** |  |  |
|  | **Urinary Bladder** | **√** |  |  |
|  | **Other urinary Organs** |  | **√** |  |
| **Eye and Orbit** | **Eye and Orbit** |  | **√** |  |
| **Endocrine system** | **Thyroid** |  | **√** |  |
|  | **Other Endocrine including Thymus** | **√** |  |  |
| **Lymphoma** | **NHL-Extranodal** |  |  | **√** |
| **Mesothelioma** | **Mesothelioma** | **√** |  |  |
